# Complementary Therapies in Allergic Rhinitis

**DOI:** 10.1155/2013/938751

**Published:** 2013-11-13

**Authors:** Ibrahim Sayin, Cemal Cingi, Fatih Oghan, Bahadir Baykal, Seckin Ulusoy

**Affiliations:** ^1^Otorhinolaryngology—Head and Neck Surgery Department, Bakırkoy Dr. Sadi Konuk Training and Research Hospital, 34147 Istanbul, Turkey; ^2^Department of Otorhinolaryngology, Faculty of Medicine, Osmangazi University, 26480 Eskisehir, Turkey; ^3^Department of ORL&HNS, Medical School, Dumlupinar University, Central Campus, Tavsanli Yolu 10 km, 43270 Kutahya, Turkey; ^4^Corlu State Hospital, Department of Otorhinolaryngology, 59850 Tekirdag, Turkey

## Abstract

*Objective*. To determine the prevalence of herbal treatment of allergic rhinitis. *Methods*. In this prospective study, patients who were diagnosed with perennial allergic rhinitis were questioned about their use of natural products/herbal therapies for their symptoms. *Results*. In total, 230 patients were enrolled. Overall, 37.3% of the patients stated that they had used natural products/herbal therapies at least once. Women were more likely than men to use herbal supplements (38.3% versus 32.4%). Ten different types of herbal supplements were identified, with stinging nettle (*Urtica dioicath*), black elderberry (*Sambucus nigra*), and *Spirulina* being the most common (12.6%, 6.1%, and 5.7%, resp.). *Conclusion*. This study found a high prevalence of herbal treatment usage for the relief of allergic rhinitis symptoms in Turkey. The herbal products identified in this study and in the literature are discussed.

## 1. Introduction

Along with acupuncture, yoga, massage, and speleotherapy, herbal therapy (HT) is a therapeutic option available as complementary alternative medicine (CAM) [1]. It has a long historical tradition, with the medicinal use of herbs being reported as early as 2800 BC. Around 400 BC, Hippocrates described the relationship between nutrition and health as “Food be medicine and medicine be food” [2].

In recent years, traditional therapies have gained in popularity for medical and economic reasons, in both developing and industrialized countries [3]. The prevalence of herbal therapy use differs according to country and medical condition. The general literature indicates that 25–50% of the general population and up to 70% of children have used a CAM method at least once [1]. A study in Americans demonstrated a 29% prevalence of HT use for rhinosinusitis [4]. In a study from Spain, 34.4% of 400 patients with allergic diseases (allergic rhinitis, asthma, and atopic dermatitis) had used at least one type of alternative medicine, and among these patients, 31.5% had used a natural medicine [5]. A recent study from England demonstrated that 65% of those questioned had used CAM for rhinosinusitis or nasal polyposis [6]. A study from Israel reported a rate of 19% for the use of CAM to treat rhinosinusitis [7].

Herbal therapies have a variety of pharmacological actions, as well as potential adverse effects and potential drug interactions. Some HTs produce bleeding, cardiovascular instability, or altered glucose levels. Others can potentiate the sedative effects of anesthesia and increase the metabolism of drugs during the postoperative period. Thus, the American Association of Anesthesia recommends that HTs be stopped 2-3 weeks before surgery [8]. 

Given the widespread and growing use of HTs and their potential pharmacodynamic and pharmacokinetic effects, an understanding of the use of HTs for various conditions would be beneficial for physicians. This idea is supported by several studies, including one that reported the need among general practitioners and allergy/immunology specialists for more knowledge about complementary medicine [9].

In our previous study regarding the prevalence of allergic rhinitis in Turkey [10], many volunteers offered information about HTs that they had used to treat their allergic rhinitis symptoms. Here, we examine the use of HTs by patients with allergic rhinitis in Eskişehir, Turkey.

## 2. Materials and Methods

This study was performed at the Department of Otorhinolaryngology, Osmangazi University Medical Faculty, Eskişehir, Turkey. Patients who were diagnosed with perennial allergic rhinitis, between January 2012 and January 2013, were asked to answer a few questions about their use of herbal treatments. The subjects who accepted were included in this study. In all patients, allergy was diagnosed based on natural history, clinical examination, and skin prick tests. At the time of diagnosis, patients were questioned about their use of herbal supplements. Pediatric patients and subjects with seasonal allergic rhinitis were not included.

The questions were the following:

1. have you used any kind of herbal treatment for your allergic condition?
2. which herbal treatment did you use?

The available literature, up to January 2013, was searched using MedLine, with the key words allergic rhinitis [AND] complementary [OR] alternative medicine [OR] herbal. All retrieved trials and experimental studies were reviewed.

## 3. Results

By the end of the 1-year period, 230 patients were enrolled. Although we had more allergy patients, not all agreed to answer questions related to complimentary treatments.

The study group, with a mean age of 34 (range, 19–49) years, consisted of 162 (70.4%) female and 68 (29.6%) male subjects. Overall, 36.6% of the subjects had used a natural product or herbal therapy at least once. Women were more likely than men to have used herbal supplements (38.3% versus 32.4%). Ten different types of herbal supplement were primarily used, with *Urtica diocia*, *Sambucus nigra*, and *Spirulina* being the most common (12.6%, 6.1%, and 5.7%, resp.). The herbal use results are summarized in Table 1.

## 4. Discussion

Turkey is a unique country; reflecting its geographic location between Europe and Asia, it combines a somewhat European culture with a strong background of Asian/oriental traditions. In a country where amulets and evil eye beads are still carried for healing, alternative medicine is of interest to many people. To date, only one reported study has addressed the use of alternative medicine by allergy patients in Turkey. That study evaluated patients with asthma, allergic rhinitis, and atopic dermatitis [11]. Among 70 patients with seasonal allergic rhinitis (SAR), the prevalence of HT use was 21.4%. The use of natural products may be related to dissatisfaction with conventional medicine, fashionable trends, media promotions, politics, or the affects of various cultures that have settled in Anatolia through history. Friends and family members also commonly influence treatment choices for chronic conditions [11]. Education, geography, and public opinion are additional contributing factors [2]. Although studies have evaluated the use of herbal supplements, the actual prevalence of their use is likely to be higher than reported. A recent survey indicated that only 43% of subjects who had used CAM had informed a doctor about this [6].

The complimentary products found to be most commonly used in our study have been extensively described in the literature.

### 4.1. Stinging Nettle (*Urtica diocia*, UD)

The leaves of the plant contain histamine, serotonin (5-hydroxytryptamine), and acetylcholine. Although it may seem inconsistent to use an extract that contains histamine to treat allergic rhinitis, subcutaneous and intravenous injections of histamine have been previously used to treat several allergic conditions such as cold urticaria with associated anaphylaxis, migraine, cluster headache associated with vasomotor rhinitis, penicillin reaction, and allergic arthritis [12]. It is also known that during allergen exposure, low plasma histamine levels, and not high plasma histamine levels, are associated with severe reactions [13]. A prospective, double-blind, comparative study of 69 allergic arthritis patients noted a significant benefit with UD versus placebo [14]. A more recent study also showed that UD inhibited proinflammatory pathways related to allergic rhinitis by antagonizing histamine 1 receptor, inhibiting prostaglandin (PG) formation and inhibiting degranulation [15].

### 4.2. *Sambucus nigra* (Elderberry)

Although this plant is commonly used in alternative treatments, to our knowledge, there is no reported study focusing on the antiallergic properties of *Sambucus nigra* extracts. Previous data for this extract have documented its use as an antiviral agent for colds, influenza, and herpes virus infections, owing to its immune-modulating and antioxidant effects [16].

### 4.3. *Spirulina*

*Spirulina* is a blue-green alga that has been commercialized as a dietary supplement for modulating immune function, as well as ameliorating various diseases [17]. A previous study in allergic rhinitis patients demonstrated that *Spirulina* can modulate the Th cell profile by suppressing the differentiation of Th2 cells. It can also inhibit the production of IL-4 [18]. In an experimental study, it was effective for relieving inflammation of the nasal mucosa and decreasing serum histamine and immunoglobulin (Ig) E levels [19]. In our double-blind, placebo-controlled study to evaluate the effectiveness and tolerability of *Spirulina* in patients with allergic rhinitis, *Spirulina* consumption significantly improved the symptoms and physical findings, including nasal discharge, sneezing, nasal congestion, and itching, compared with placebo [17].

### 4.4. Butterbur (*Petasites hybridus*)

Butterbur has been extensively studied, especially related to its beneficial effects in allergic rhinitis. In a comparative, randomized study in 125 patients with SAR, both butterbur (butterbur-carbon dioxide extract tablets, Ze 339; 1 tablet, 4 times/day; *n* = 61) and cetirizine (1 tablet each evening; *n* = 64) were effective in improving SF-36 and global improvement scores [20]. However, sedative effects were observed in the cetirizine group. Similar results were obtained in a more recent study comparing the efficacy of butterbur (Ze 339; 8 mg total petasin; 1 tablet, 3 times/day), fexofenadine (180 mg once/day), and placebo in 330 patients [21]. Butterbur and fexofenadine were comparably effective, compared with placebo. Another study demonstrated that butterbur (Ze 339; 2 tablets, 3 times/day for 1 week) improved day and nighttime nasal symptoms, decreased nasal resistance as verified by rhinomanometry, and decreased anti-inflammatory mediators (histamine, LTB4, and cysteinyl-LT) in nasal fluids and serum [22]. Petasins (petasin, isopetasin, and neopetasin) are believed to be the pharmacologically active components of butterbur extracts [23]. A randomized, placebo-controlled study demonstrated that in sensitized patients, butterbur protected against adenosine monophosphate-induced nasal responsiveness during grass pollen season [24]. In contrast, a more recent study found no significant effect of butterbur (50 mg twice daily for 2 weeks) on peak nasal inspiratory flow, total nasal symptom score, eye symptom score, or quality of life in patients with intermittent allergic rhinitis, compared with placebo [25]. Dose-dependent effects were investigated in 186 patients with intermittent allergic rhinitis who were treated with butterbur Ze 339 (high dose: 1 tablet 3 times/day, *n* = 60; low dose: 1 tablet twice daily, *n* = 65) or placebo (*n* = 61) for 2 weeks [26]. Significant improvement of symptoms was observed with the use of butterbur, which showed a positive dose-dependent effect and side effects similar to those of the placebo. In an experimental study, an aqueous ethanol extract of the aerial parts of Japanese butterbur (JBE) directly suppressed smooth muscle contraction and inhibited cutaneous anaphylactic reactions; LT C4, D4, and E4 synthesis; TNF-*α* production; and mast cell degranulation [27]. An open postmarketing surveillance study on 580 patients (Ze 339; 2 tablets/day for 2 weeks) showed significant improvement in rhinorrhea, sneezing, nasal congestion, itchy eyes and nose, red eyes, and skin irritation associated with SAR in 90% of the patients [28]. An antiallergic comedication administered to 44% of the patients did not give a better result than that with Ze 339 monotherapy. Adverse events occurred at a rate of 3.8% in the study.

### 4.5. Lemon Peel

There is only one case report demonstrating the effectiveness of lemon peel in allergic rhinitis [29]. However, it has been noted that the peels of citrus fruits (grapefruit, lemon, lime, and orange) contain high levels of phenolics, flavonoids, ascorbic acid, and carotenoids, which have antioxidant properties [30].

### 4.6. *Allium cepa* (Onion)

One experimental study has shown the antiallergic effects of *Allium cepa*. Although it has potential antihistamine, anti-inflammatory, and antioxidant activities [31], no study has addressed its use in allergic rhinitis.

### 4.7. Honey

In Turkey, honey is believed to be a beneficial supplement for various conditions. A previous study in 304 asthmatic families demonstrated that 26% of parents had previously used Turkish wild honey for their children's disease [32]. Only one study of 36 patients exists in the literature, and it did not find honey to be effective in allergic rhinitis, compared with placebo [33].

### 4.8. *Prunus dulcis* (Almond)/*Petroselinum crispum* (Parsley)

Although some participants noted the use of these natural products, there are no reported studies addressing their use in allergic rhinitis.

A review of the literature on herbal treatments for the symptoms of allergic rhinitis revealed studies on the medicinal benefits of many more herbs and plants.

### 4.9. *Moringa oleifera* (MO) Lam

MO Lam. is a traditional herbal therapy in South Asia. The flowers, roots, seeds, and fruits of the plant are used for herbal therapy. The plant contains many important dietary elements such as proteins, minerals, and vitamins. More than 100 studies evaluating the effectiveness of this extract in various conditions have been reported, documenting its antioxidant, anticancer, antidiabetic, antihypertensive, antiepileptic, and cholesterol-lowering benefits [34]. Potential antiallergic effects of MO have been demonstrated in an experimental study [35]. MO extract inhibited inducible anaphylactic shock, passive cutaneous anaphylaxis activated by anti-IgE antibody, and histamine release from mast cells.

### 4.10. *Astragalus membranaceus* (AM)

Although more than 400 studies have been reported, only one focused on the effects of AM in SAR. This placebo-controlled, double-blind study revealed that the use of AM had a beneficial effect in SAR [36]. In 48 subjects who had moderate to severe SAR, AM significantly decreased the intensity of rhinorrhea; however, the effects of AM on other symptoms did not differ from those of the placebo. Of note, AM was effective against SAR due to weed pollen allergy in this study.

### 4.11. *Flos magnolia* (FM)

FM is reported to have anti-inflammatory, antimicrobial and antiallergic activities [37]. Different FM species, which include *Magnolia biondii*, *M. denudata*, *M. kobus*, *M. liliiflora*, *M. sargentiana*, and *M. sprengeri*, exhibit different pharmacological properties. Ethanol extracts of FM produce a concentration-dependent inhibition of compound 48/80-induced histamine release and anti-inflammatory and antimicrobial activities [37].

### 4.12. Gyokuheifusan (GHS; Astragalus Root, Atractylodes Rhizome, and Saposhnikovia Root Combination)

The potential usefulness of GHS in Japanese cedar pollen-induced allergic rhinitis has been demonstrated in guinea pigs [38]. In this murine model of antibody production, GHS significantly increased the concentration of ovalbumin-specific immunoglobulin in mice that were sensitized intraperitoneally to ovalbumin and upregulated the immune response in mice previously exposed to the antigen. In contrast, GHS significantly decreased ovalbumin-specific immunoglobulin in mice that were intranasally sensitized to the antigen. The authors suggested that GHS may consolidate the resistance of the nasal mucosa to protect against ovalbumin invasion, resulting in suppression of ovalbumin-specific antibodies in serum.

### 4.13. Biminne

This Chinese herbal formula consists of 11 different ingredients, including extracts of *Scutellaria baicalensis* (Baikal or Chinese skullcap), *Ginkgo biloba* (ginkgo), *Epimedium sagittatum* (yin yang huo or horny goat weed), and *Schizandra chinensis* (wu wei tzu or schizandra) and the pulp of *Prunus Mume* (Japanese apricot), *Ledebouriella divaricata* (Fang feng), and *Astragalus membranaceus* (astragalus). A randomized, double-blind, placebo-controlled study in 58 subjects demonstrated a beneficial effect on some allergic rhinitis symptoms and a decreased total serum IgE level [39]. Although the therapy period was 12 weeks, the beneficial effects persisted for 1 year. Another study reported that the frequency of sneezing and nasal rubbing was decreased with the use of biminne in mice in which an allergic rhinitis model was induced by intraperitoneal injection of ovalbumin; the serum total and ovalbumin-specific IgE levels were also decreased [35]. Biminne has also been shown to alter vascular permeability in the nasal mucosa of sensitized rats [40]. A more recent experimental study indicated that biminne therapy could downregulate Th2 cells [41]. Two relevant cytokines, interleukin-(IL-) 4 and IL-5, were also downregulated, whereas Th1 cells and interferon-*γ* were upregulated. It was suggested that altering the Th2/Th1 ratio may explain the therapeutic effect of biminne in airway allergies.

### 4.14. RCM-101

In a previous randomized, placebo-controlled study, RCM-101 reduced SAR symptoms [42]. Possible tissue effects were evaluated in more recent studies. An experimental study demonstrated that RCM-101 inhibited the compound 48/80-induced release of histamine, PGE2, and LTB4; RCM-101 also inhibited the expression of cyclooxygenase-(COX-) 2 protein, whereas COX-1 expression was not affected. [43]. More recently, possible multiple inhibitory actions of RCM-101 against inflammatory mediators have been described [43]. RCM-101 inhibited L-arginine-induced relaxation in endothelium-denuded rat aortic preparations and inhibited PGE2 and nitric oxide (NO) production in Raw 264.7 cells. Furthermore, RCM-101 and some of its ingredients (e.g., Radix glycyrrhizae, Radix bupleuri, Radix saposhnikoviae, and Atractylodis rhizoma macrocephalae) also inhibited NO production and inducible-NO synthase (NOS) protein expression in Raw 264.7 cells [44].

### 4.15. RCM-102

An experimental study showed the anti-inflammatory effects of RCM-102 [45]. Intraperitoneal use of this herbal formula significantly decreased compound 48/80-induced histamine release, NO and PGE2 production, and INOS and COX-2 expression.

### 4.16. Shi-Bi-Lin (SBL)

This is a modified form of the classic Chinese formula Jia Wei Cang Er Zi San, [46] which has been used to treat allergic rhinitis, chronic rhinitis, and sinusitis for several centuries [47]. In a double-blind, placebo-controlled, randomized study with 126 subjects, SBL significantly decreased nasal blockage and enhanced some quality of life scores [48]. The effect of SBL lasted for 2 weeks after treatment, and no side effects were observed. In cell cultures, SBL significantly inhibited mast cell-derived IL-4 and TNF-*α* secretion, whereas IL-8 release and mRNA expression of these cytokines were not changed [46]. An experimental study in guinea pigs showed that SBL can suppress the production of IgG1 and decrease thromboxane B2 levels in nasal lavage fluid; the levels of histamine and peptide leukotrienes were not altered, but eosinophil infiltration and endothelial NOS immune reactivity were decreased in the nasal tissue [46].

### 4.17. Dishen Qufeng Decoction (DSQFD)

A randomized clinical trial compared the effectiveness of DSQFD with that of cetirizine in 60 patients [49]. The total response rates to DSQFD and cetirizine were 83.3% and 86.7%, respectively, and both effectively decreased the peripheral blood eosinophil count.

### 4.18. Hochuekkito (HET)

A randomized, placebo-controlled study in 60 patients demonstrated that HET significantly improved nasal symptomatic scores. HET also suppressed total serum IgE as well as IL-4-stimulated production of PGE2 and leukotriene C4 and IL-4-stimulated expression of COX-2 mRNA in polymorphonuclear neutrophils [50].

### 4.19. *Lycopus lucidus* (LAE)

LAE is an oriental herbal extract used in Republic of Korea. LAE has been shown to inhibit mast cell-derived, immediate-type allergic reactions [51]. In this study, LAE inhibited compound 48/80-induced systemic reactions in mice. It also had an inhibitory effect on proinflammatory cytokines, p38 mitogen-activated protein kinase, and nuclear factor-kappaB (NF-*κ*B). 

### 4.20. Aller-7/NR-A2

This polyherbal product is a mixture of seven herbal extracts (*Phyllanthus emblica*, *Terminalia chebula*, *Terminalia bellerica*, *Albizia lebbeck*, *Piper nigrum*, *Zingiber officinale*, and *Piper longum*). Its antioxidant [52], anti-inflammatory [53], antihistamine, antispasmodic, and mast cell stabilization activities [54] have been demonstrated in previous experimental studies. In a safety study performed according to the guidelines of the Organization for Economic Cooperation and Development, World Health Organization and US Environmental Protection Agency, no adverse effects and no fetal or maternal teratogenic effects were observed [55]. In another safety study, the “no observed adverse effect level” (NOAEL) of Aller-7 was greater than 1,000 mg/kg body weight [55]. A multicenter clinical trial demonstrated the efficacy of Aller-7 in 545 patients with allergic rhinitis, as sneezing, rhinorrhea, and nasal congestion were significantly improved [56]. Mucociliary clearance time, peak nasal inspiratory and expiratory flow rates, and absolute eosinophil count were also improved. No major, but some minor, side effects were observed with treatment.

### 4.21. Gamma-Tocopherol (GammaT)

GammaT is the primary form of dietary vitamin E. In rats, acute gammaT therapy for 4 days blocked eosinophil infiltration into airspaces and tissues of the nose, sinus, and nasolacrimal duct [57]. Furthermore, gammaT decreased allergen-induced mucous cell metaplasia, increased goblet cell number, and amount of intraepithelial mucus storage in the nasal airway.

### 4.22. Gall of *Rhus javanica* (GRJ)

A possible antianaphylactic effect was demonstrated for GRJ in an experimental study on mice, rats, and human mast cells [58]. GRJ inhibited compound 48/80-induced systemic reactions and decreased IgE-mediated local allergic reactions. Additionally, GRJ inhibited histamine release from rat peritoneal mast cells activated by compound 48/80 or IgE, in a dose-dependent manner. This effect was mediated by modulation of cAMP and [Ca^2+^]_*i*_ in mast cells. GRJ also decreased TNF-*α* and IL-6 secretion in human mast cells.

### 4.23. *Houttuynia cordata* Water Extract (HCWE)

HCWE is a traditional herbal therapy with known antioxidant and anticancer activities. In mice, oral HCWE inhibited compound 48/80-induced systemic anaphylaxis. It also decreased compound 48/80-induced mast cell degranulation and colchicine-induced deformation of rat peritoneal mast cells (RPMCs). Additionally, HCWE inhibited histamine release and calcium uptake by RPMCs induced by compound 48/80 or anti-DNP IgE, in a dose-dependent manner. Thus, HCWE may be beneficial in the treatment of mast cell-mediated anaphylactic responses [59].

### 4.24. Moutan Cortex Radicis (MCR)

MCR reduced the expression and secretion of eotaxin, a potent eosinophil-specific chemokine, inhibited eosinophil migration toward A549 medium, and suppressed the activation of NF-*κ*B [60].

### 4.25. Xinqin

In allergic rhinitis induced by 4-toluene diisocyanate, xinqin tablets reduced symptoms of nasal hypersensitivity, decreased histamine release from the nasal mucosa, and inhibited NOS activity [61].

### 4.26. Kebimin Decoction (KD)

In a study comparing KD and Xinfang rhinitis capsules, KD reduced the level of adhesion molecules and altered the Th1/Th2 balance, by decreasing Th1 cytokine levels and increasing Th2 cytokine levels, in allergic rhinitis patients [62]. 

### 4.27. Gamibojungikgitang (GBIT)

GBIT, an oriental herbal extract, has been used to treat allergic rhinitis in Republic of Korea, China, and Japan. In an experimental study, GBIT inhibited compound 48/80-induced systemic anaphylactic shock in a dose-dependent manner [63]. Furthermore, GBIT pretreatment of rat peritoneal mast cells decreased compound 48/80-induced histamine release, in a dose-dependent manner. GBIT inhibited IL-6 secretion induced by phorbol 12-myristate 13-acetate (PMA) and a calcium ionophore (A23187) in human mast cells and inhibited passive cutaneous anaphylaxis activated by antidinitrophenyl IgE.

### 4.28. *Isodon japonicus* Hara (IJAE)

The herbal extract IJAE has been used in Republic of Korea for its anti-inflammatory effects. In mice, IJAE inhibited compound 48/80-induced systemic reactions and decreased IgE-mediated local allergic reactions. Expression of TNF-*α* mRNA in HMC-1 cells was also inhibited by IJAE. Additionally, IJAE inhibited histamine release from rat peritoneal mast cells activated by compound 48/80 or IgE in a dose-dependent manner. GRJ also decreased TNF-*α* secretion in human mast cells [64]. In a previous study, it was shown that the extract inhibited IL-6 secretion induced by PMA and A23187 in human mast cells [65]. 

### 4.29. Bishudiwan

In an experimental allergic rhinitis model, bishudiwan was effective in relieving allergic rhinitis symptoms by decreasing serum IgE levels and decreasing nasal and serum histamine release [66].

### 4.30. Ryokan-kyomi-shinge-nin-to (RKS)

The oriental herbal extract RKS is used in Japan. In an experimental study, RKS was shown to decrease sneezing and inhibit vascular permeability induced by histamine or serotonin, although it did not affect histamine release [67].

### 4.31. Sho-seiryu-to (SST)

The major effective ingredient in SST is ephedrine [68]. SST has been shown to inhibit the secretion of water and electrolytes from the nasal glands, through an anticholinergic effect [69]. In a nasal allergy model in rats, SST had anti-type 1 allergy activity [70]. Among 914,612 Taiwanese subjects diagnosed with allergic rhinitis, 35.6% had taken Chinese herbal medicines [4] and SST was found to be one of the two most commonly used combination treatments. In an experimental study, the numbers of sneezes and scratches were significantly reduced by pretreatment with SST [71]. Eosinophil infiltration and volume changes in the nasal mucosa induced by LT D4 were also inhibited. Another study demonstrated that SST could inhibit allergen-induced synthesis of TNF-*α*, IgE, and IL-10, while levels of IL-5, IL-6, and IFN-*γ* did not change [72]. SST may also suppress IL-4 production in CD4+ T cells by influencing CD28-CD86 interactions [70] and may correct a Th2-dominant condition in allergic rhinitis [73].

### 4.32. Shin-yi-san + Xiao-qing-long-tang + Xiang-sha-liu-jun-zi-tang Mixture

A study in allergic rhinitis patients showed that a 3-month treatment with this combination suppressed inflammation in the nasal mucosa [74]. The treatment normalized the stimulatory effects of allergic nasal discharge in patients with high-IgE allergic rhinitis. In another study, treatment with this combination decreased serum total and house dust mite-specific IgE levels [74]. Additionally, IL-10 levels were increased, while IL-5 and IFN-*γ* production and COX-2 mRNA expression were decreased after treatment.

### 4.33. Grapeseed Extract

An 8-week, randomized, double-blind, placebo-controlled study of treatment with grapeseed extract demonstrated no significant improvement in symptoms in SAR [75].

### 4.34. Tongkyutang (TKT)

In a mouse model, TKT inhibited compound 48/80-induced histamine release from rat peritoneal mast cells, in a dose-dependent manner [76]. Oral administration of TKT also inhibited the passive cutaneous anaphylactic reaction induced by antidinitrophenyl IgE and IL-1*β* and TNF-*α* secretion induced by PMA and A23187.

### 4.35. Shosaiko-to (ST)

Oral administration of ST significantly inhibited histamine release, in a dose-dependent manner [77]. However, the extract failed to inhibit compound 48/80-induced histamine release in mast cells. The authors suggested that ST inhibited IgE receptor-associated protein phosphorylation in the histamine release pathway.

### 4.36. Biminkang Mixture (*Artemisia rupestris* L)

This extract is used primarily as a Kazak folk herb. When 162 patients with allergic rhinitis were treated with the extract and 52 subjects received a placebo, the total effectiveness and recurrence rates for the treatment versus control groups were 93.9 versus 69.2% and 46.8 versus 87.1%, respectively [78].

### 4.37. Tian-huang-ling Granule (Larvae of a Silkworm with Batrytis, Milk Vetet, and Long-Spur Epimedium)

In a study of 60 cases of allergic rhinitis, 96.6% of the subjects demonstrated symptomatic improvement [79]. No adverse effect was seen, and the results were better than those in subjects who received ketotifen. Additionally, total eosinophil and basophil blood counts were significantly decreased after the treatment.

### 4.38. Bu Qi Gu Biao

In total, 500 subjects with allergic rhinitis have been treated with this extract since 1980 [80]. A long-term curative effect was observed in 87% of the patients. Of these subjects, 200 underwent assessments that indicated the therapy could improve immune function and cyclic nucleotide metabolism. Blood flow in nasal mucosal tissue was improved as were cell shape and function.

## 5. Conclusion

There is growing worldwide interest in the use of herbal supplements for many indications. Some herbal treatments contain well-known active ingredients, which are known and accepted by modern medicine. Our examination of the prevalence of herbal treatments for allergic rhinitis in Turkey demonstrated that a high percentage of patients have used these products to relieve the symptoms of allergic rhinitis. These data may be unique to our country owing to its culture and tradition. For example, some products such as honey, onion, and parsley are believed to be healthy and curative and thus are widely used for many diseases. Increased use of these products in developed countries may be influenced more by hope than history.

Additional studies would help to clarify the active ingredients in herbal supplements used to treat allergic rhinitis and may lead to the development of new drugs from herbal extracts. Nevertheless, the placebo effect is a major issue that must always be considered with regard to the effectiveness of complimentary treatment modalities.

## Figures and Tables

**Table 1:** The prevalence of the herbal use among the study group.

Herbal type	Male	Female	Total
*Urtica dioica*	7 (3%)	22 (9.6%)	29 (12.6%)
*Sambucus nigra* (Elderberry)	3 (1.3%)	11 (4.8%)	14 (6.1%)
*Spirulina*	5 (2.1%)	8 (3.5%)	13 (5.7%)
Honey	4 (1.7%)	6 (2.6%)	10 (4.3%)
Butterbur	1 (0.4%)	4 (1.7%)	5 (2.1%)
*Allium cepa* (onion)	None	4 (1.7%)	4 (1.7%)
*Prunus dulcis* (almond)	1 (0.4%)	3 (1.3%)	4 (1.7%)
*Petroselinum crispum* (parsley)	1 (0.4%)	2 (0.9%)	3 (1.3%)
Lemon peel	None	2 (0.9%)	2 (0.9%)
Total	22/68 (32.4%)	62/162 (38.3%)	84/230 (36.6%)
